# Candidate Causal Regulatory Effects by Integration of Expression QTLs with Complex Trait Genetic Associations

**DOI:** 10.1371/journal.pgen.1000895

**Published:** 2010-04-01

**Authors:** Alexandra C. Nica, Stephen B. Montgomery, Antigone S. Dimas, Barbara E. Stranger, Claude Beazley, Inês Barroso, Emmanouil T. Dermitzakis

**Affiliations:** 1 Wellcome Trust Sanger Institute, Wellcome Trust Genome Campus, Hinxton, Cambridge, United Kingdom; 2 Department of Genetic Medicine and Development, University of Geneva Medical School, Geneva, Switzerland; 3 Harvard Medical School/Brigham and Women's Hospital, Boston, Massachusetts, United States of America; Georgia Institute of Technology, United States of America

## Abstract

The recent success of genome-wide association studies (GWAS) is now followed by the challenge to determine how the reported susceptibility variants mediate complex traits and diseases. Expression quantitative trait loci (eQTLs) have been implicated in disease associations through overlaps between eQTLs and GWAS signals. However, the abundance of eQTLs and the strong correlation structure (LD) in the genome make it likely that some of these overlaps are coincidental and not driven by the same functional variants. In the present study, we propose an empirical methodology, which we call Regulatory Trait Concordance (RTC) that accounts for local LD structure and integrates eQTLs and GWAS results in order to reveal the subset of association signals that are due to *cis* eQTLs. We simulate genomic regions of various LD patterns with both a single or two causal variants and show that our score outperforms SNP correlation metrics, be they statistical (r^2^) or historical (D'). Following the observation of a significant abundance of regulatory signals among currently published GWAS loci, we apply our method with the goal to prioritize relevant genes for each of the respective complex traits. We detect several potential disease-causing regulatory effects, with a strong enrichment for immunity-related conditions, consistent with the nature of the cell line tested (LCLs). Furthermore, we present an extension of the method in *trans*, where interrogating the whole genome for downstream effects of the disease variant can be informative regarding its unknown primary biological effect. We conclude that integrating cellular phenotype associations with organismal complex traits will facilitate the biological interpretation of the genetic effects on these traits.

## Author Summary

Genome-wide association studies have led to the identification of susceptibility loci for a variety of human complex traits. What is still largely missing, however, is the understanding of the biological context in which these candidate variants act and of how they determine each trait. Given the localization of many GWAS loci outside coding regions and the important role of regulatory variation in shaping phenotypic variance, gene expression has been proposed as a plausible informative intermediate phenotype. Here we show that for a subset of the currently published GWAS this is indeed the case, by observing a significant excess of regulatory variants among disease loci. We propose an empirical methodology (regulatory trait concordance—RTC) able to integrate expression and disease data in order to detect causal regulatory effects. We show that the RTC outperforms simple correlation metrics under various simulated linkage disequilibrium (LD) scenarios. Our method is able to recover previously suspected causal regulatory effects from the literature and, as expected given the nature of the tested tissue, an overrepresentation of immunity-related candidates is observed. As the number of available tissues will increase, this prioritization approach will become even more useful in understanding the implication of regulatory variants in disease etiology.

## Introduction

The biological interpretation of genome-wide association study (GWAS) signals [1]–[5] is very challenging since most candidate loci fall either in gene deserts or in regions with many equally plausible causative genes. Following the concurrent progress in understanding the genetic basis of regulatory variation [6]–[9], differential gene expression has been proposed as a promising intermediate layer of information [10] to aid this interpretation [11]. Most commonly, interrogating the GWAS SNPs themselves for significant associations with gene expression [12]–[13] has been employed to explain some of the GWAS results. However, the ubiquity of regulatory variation throughout the human genome [6],[14] makes coincidental overlaps of eQTLs and complex trait loci very likely. This likelihood is a direct consequence of the correlation structure in the genome (linkage disequilibrium - LD), which makes functionally unrelated variants statistically correlated.

As sample sizes increase, allowing the discovery of larger numbers of eQTLs of smaller effect size and as the expression experiments will be performed in a larger variety of tissues, we can envisage that almost every gene will have an associated eQTL under a certain condition. Consequently, the probability that any of these will map to a genomic region where a GWAS SNP also resides is very high. Therefore, it is important to emphasize that while it is very tempting to infer potential causal mechanisms based on such overlaps, this would be a naïve inference in the absence of additional supporting evidence for causality. In the long run, this will not only be an issue for gene expression, but also for any other cellular phenotype. Association studies for intermediate phenotypes with possible relevance to complex traits are underway and their results will overlap some of the GWAS signals. The biological meaning of these overlaps will again need to be evaluated in the context of the genome's correlation structure.

It is not evident though how to model each genomic region with overlapping association signals in the absence of information about the history of the region. Accounting for the historical parameters of a region under the coalescent, while desirable, is computationally and practically not feasible since the human population history is too complex to properly model and small deviations or slightly incorrect assumptions could create false signals or reduce power.

In order to distinguish such accidental colocalizations [15]–[16] from true sharing of causal variants, we propose here an empirical methodology instead. This directly combines eQTL and GWAS data while accounting for the LD of the region harbouring the GWAS SNP. We demonstrate the value of the approach by predicting the regulatory impact of several GWAS variants in *cis* and *trans* and we also show that the correlation strength (r^2^, D') between the GWAS SNP and the eQTL is not a sufficient predictor of regulatory mediated disease effects.

## Results

### Current GWAS signals are enriched for regulatory variants

To identify likely causal effects (not variants since we do not have full sequencing data) associated with complex traits and diseases we took advantage of published association data catalogued in the NHGRI [17] database and gene expression data generated in LCLs derived from HapMap 3 individuals (see Methods). In this study, we limited the expression analysis to the 109 CEU individuals, as they are the closest in ancestry to the majority of individuals in published GWAS studies. We used the NHGRI database (accessed 02.03.09) to extract 976 GWAS SNPs with minor allele frequency (MAF) >5% that were also genotyped in the HapMap 3 CEU, thus allowing to test the exact GWAS SNPs for associations with differential gene expression in LCLs. In total we examined 17673 genes. In order to discover eQTLs, we used Spearman Rank Correlation (SRC). This method [14] captures the vast majority of associations discovered with standard linear regression (LR) models, with the additional advantage that it's not affected by outliers and hence has more power and allows direct comparison of nominal P-values. We looked for both proximal (*cis*) and distal (*trans*) effects as follows: variants within 1Mb on either side of the transcription start site (TSS) of a gene are considered to be acting in *cis*, while those at least 5 Mb downstream or upstream of the TSS or on a different chromosome are considered to be acting in *trans*.

**Figure 1:**
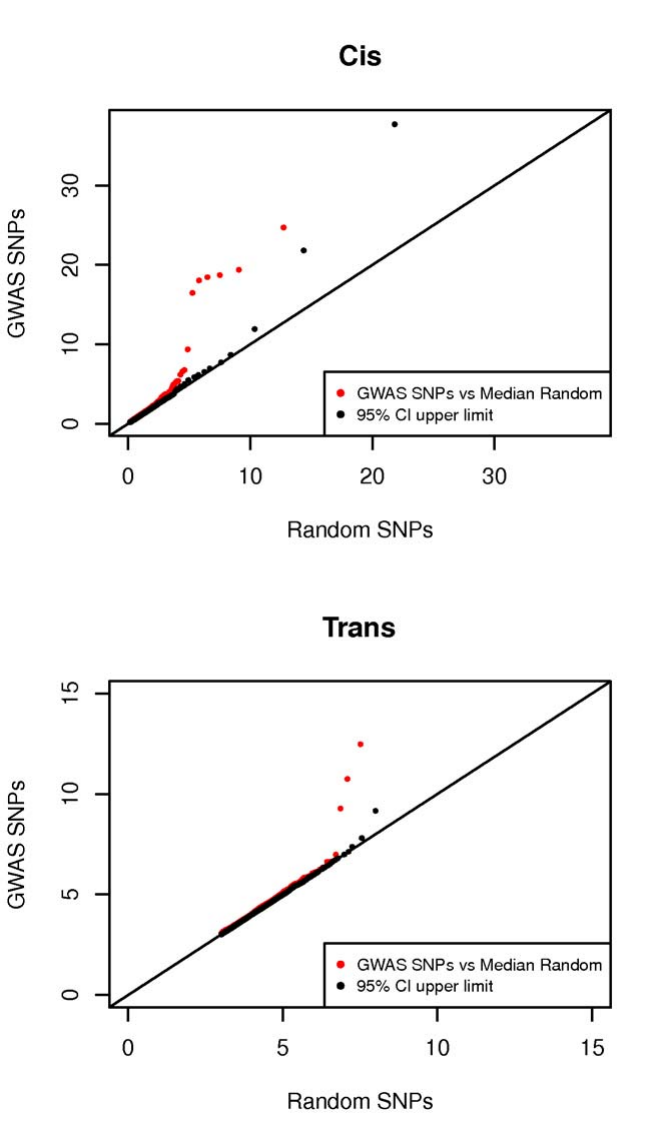
Excess of regulatory variants among GWAS signals. QQ plot depicting the excess of significant regulatory signal in GWAS data (976 NHGRI SNPs). For both the *cis* and *trans* analyses, the −log_10_(P-value) of the best associations per SNP are plotted. In red, the distribution of these values for GWAS SNPs is compared to that of the median of 1,000 sets of 976 random SNPs with same MAF distribution. In black, the estimated upper limit of the 95% confidence interval is plotted.

**Figure 2:**
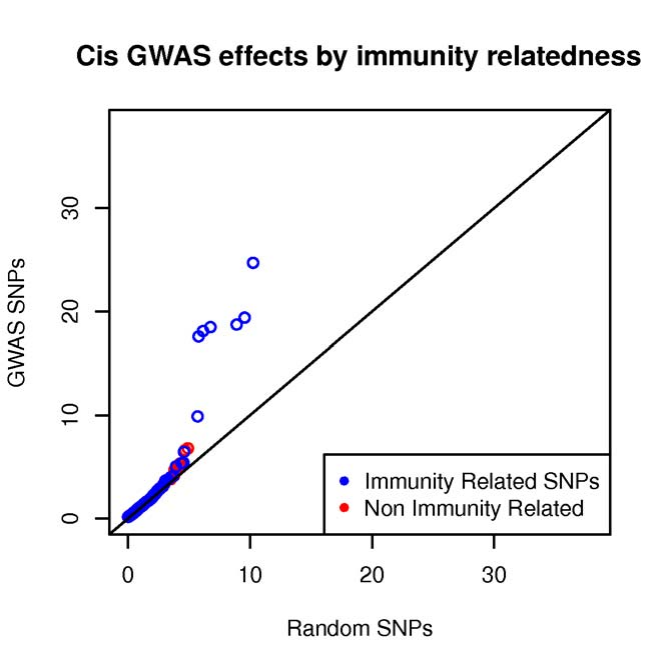
*Cis* regulatory enrichment stratified by immunity relatedness. The −log10(P-value) of the best associations per GWAS SNPs and a set of random SNPs are plotted. As expected given the tissue (LCLs), immunity related phenotypes are mainly responsible for the enrichment.

In order to assess the overall impact of the currently known GWAS SNPs on expression, we contrasted their *cis* and *trans* effects to those of a random set of SNPs, representing the null. In a QQ plot (Figure 1), we compare the distributions of the best *cis* and *trans* association p-values per SNP for the 976 GWAS SNPs (observed) to 1000 sets of most significant p-values of 976 random SNPs each (expected). The 1000 random sets of 976 SNPs were sampled to have identical MAF distribution to the GWAS SNPs. In *cis*, we observe a much stronger regulatory signal in the GWAS data compared to random (Figure 1). The significant difference between the two becomes apparent above a −log_10_(P-value) = 4. In *trans*, we also detect a more significant regulatory signal for GWAS SNPs compared to random, however not as strong as in *cis*. This is to be expected given that the much greater statistical space we're exploring in *trans* limits the power to detect such effects. Nevertheless, despite their confinement to one tissue type - LCLs, these comparisons support the overall explanatory potential of regulatory variation for the biological effects of GWAS variants. As expected given the tissue nature, the phenotypes responsible for this enrichment are immunity related (Figure 2).

### RTC score to distinguish between causal effects and coincidental overlaps

To identify the subset of causal effects from the regulatory enrichment observed, we focused only on the genomic regions harbouring either *cis* or *trans* eQTLs. We split the genome into recombination hotspot intervals based on genome-wide estimates of hotspot coordinates from McVean et.al. [18] Limiting the search space for causal effects to these intervals is a reasonable conventional approach, as few or no recombination events are expected between the reported associated SNPs and the functional variants they are tagging.

Given the abundance of *cis* eQTLs in the genome, mere interval overlap in not sufficient to claim that a colocalized *cis* eQTL and a GWAS SNP are tagging the same functional variant. However, if the GWAS SNP and the eQTL do tag the same causal SNP, we expect that removing the genetic effect of the GWAS SNP will have a marked consequence on the eQTL association. Starting from this hypothesis, we developed an empirical method to uncover regulatory mediated associations with complex traits. For all genes with a significant *cis* eQTL (0.05 permutation threshold as defined in Stranger et.al. 2007, see Methods) in a given interval, we create corrected phenotypes from the residuals of the standard LR of the GWAS SNP against normalized expression values of the gene for which we have an eQTL. The residuals capture the remaining unexplained expression variance after the removal of the GWAS SNP effect. We redo the SRC analysis with the pseudo phenotype and retain the adjusted association P-value.

Depending on the internal LD structure of the hotspot interval, the correlation between the GWAS SNP and the eQTL will vary, hence so will the P-values after and before correction. One way to assess the relevance of the GWAS SNP to the eQTL is to compare its correction impact to that of all other SNPs in the interval. For this purpose, we define a Regulatory Trait Concordance (RTC) Score for each gene-GWAS SNP combination as follows, taking into account the ranking of the correction with respect to all SNPs in the interval (Rank_GWAS SNP_) and the total number of tested SNPs (N_SNPs_).
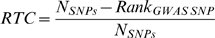



The rank denotes the number of SNPs which when used to correct the expression data, have a higher impact on the eQTL (smaller adjusted P-value) than the GWAS SNP (i.e. Rank*_GWAS SNP_* = 0 if the GWAS SNP is the same as the eQTL SNP, Rank*_GWAS SNP_* = 1 if of all the SNPs in the interval, the GWAS SNP has the largest impact on the eQTL). Given this, the RTC Score will always be in the range (0,1], with values close to 1 indicating that the GWAS effect is the same as the eQTL effect.

### RTC properties under different simulation scenarios

We investigated the properties and robustness of the RTC score under the null hypothesis (H_0_: eQTL and GWAS are tagging two different causal SNPs) and the alternative hypothesis (H_1_: same causal SNP). For this purpose, we have simulated causal SNPs (cSNP), eQTLs and dSNPs (see Methods) varying the LD levels between them as well as the LD pattern of the hotspot interval where they reside. We have then masked the cSNPs and calculated the RTC score under these different LD scenarios for both hypotheses.

**Figure 3:**
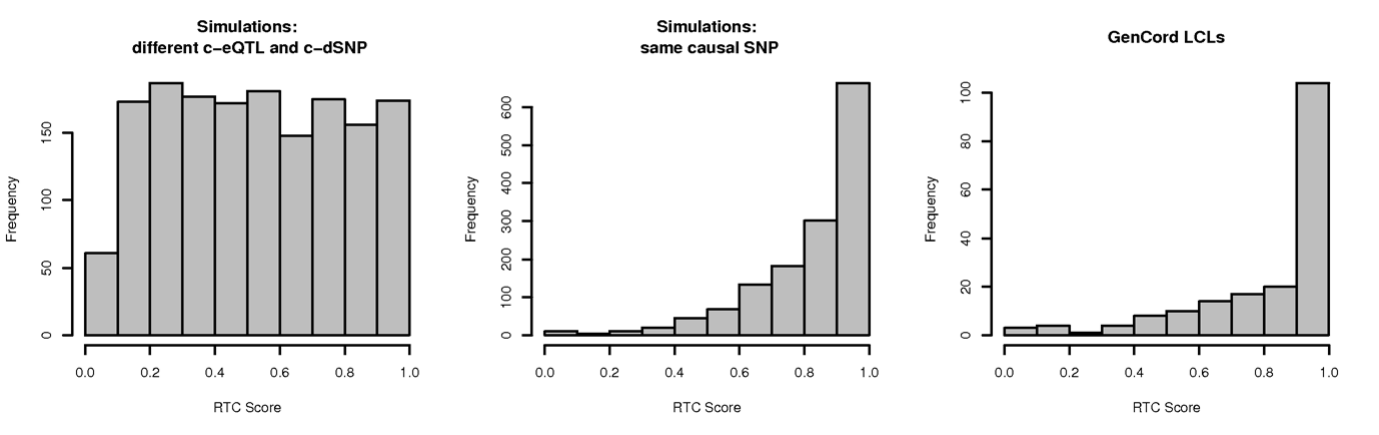
RTC score distribution. The RTC score is uniformly distributed for simulated eQTLs and dSNPs tagging two different causal variants in the same interval (left panel). The RTC Score is right-skewed for simulated eQTLs and dSNPs tagging the same functional variant (middle panel). The RTC score is sensitive to associations tagging a common functional variant in non-simulated data, when the GWAS trait is gene expression (GenCord LCL samples – right panel).

The RTC score is uniformly distributed under the null, when the simulated causal eQTL SNP (c-eQTL) and the causal disease SNP (c-dSNP) are different (Figure 3, left panel).

Under the H_1_ on the other hand, the RTC score is right skewed, with a clear enrichment for values close to 1 recovering the single causal SNP effect (Figure 3, middle panel).

The simulations show that the complexity and variability of the LD structure in the genome impede the simple use of correlation metrics to infer shared causal effects.

**Figure 4:**
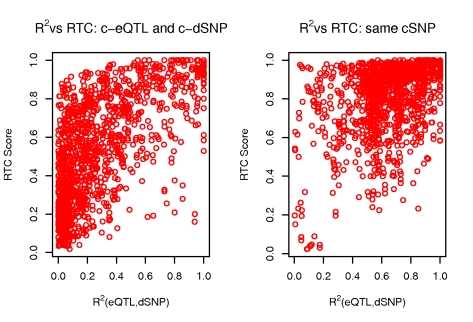
Properties of the RTC score when varying r^2^. Simulation results depicting the relationship between the RTC score and the r^2^ (eQTL, dSNP) when they tag different causal SNPs (H_0_: left panel) versus one causal SNP (H_1_: right panel). The RTC increases as expected with increased r^2^ between the eQTL and the dSNP, but when tagging the same functional variant, various lower pairwise r^2^ combinations can determine a high RTC. This makes r^2^ on its own insufficient to detect shared causal effects.

**Figure 5:**
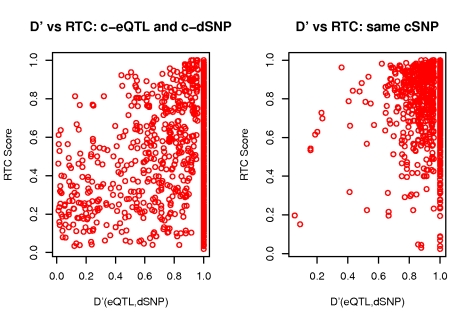
RTC score properties when varying D'. Simulation results depicting the relationship between the RTC score and the D' (eQTL, dSNP) when they tag different causal SNPs (H_0_: left panel) versus one causal SNP (H_1_: right panel). D' is not correlated with RTC, therefore it will not determine high scores on its own in the absence of a common functional variant. Under the H_1_, the majority of high RTC scoring pairs have high D', but in the case of a perfect historical correlation scenario, it's impossible to distinguish causal from coincidental effects with D' only.

The statistical correlation (r^2^) between the eQTL and the dSNP is not on its own sufficient to predict whether they tag the same cSNP (Figure 4). The RTC outperforms r^2^ as it is able to recover causal effects even for low correlated pairs. The historical correlation metric between eQTLs and dSNPs (D') is also not fully predictive of high RTC scores (Figure 5). We observe from the H_0_ simulation results that D' is not correlated with RTC, meaning that when the eQTL and dSNP tag different functional variants, the RTC score is not high just because D' is high. In addition, while high RTC scoring cases cluster much tighter around high D' values under the H_1_ compared to r^2^ previously, a high D' is not sufficient to predict causal effects. That is because it would be impossible to distinguish causal from coincidental effects given a perfect historical correlation scenario.

**Figure 6:**
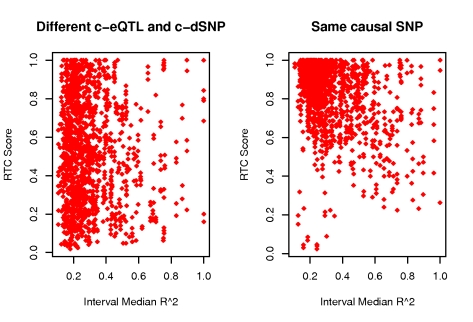
RTC score properties when varying the median r^2^ of the hotspot interval. Simulation results depicting the relationship between the RTC score and the local LD structure (median r^2^) under the null (different causal SNPs - left panel) and alternative hypothesis (same causal SNP - right panel). Under H_0_, the RTC score is evenly distributed, therefore intervals with overall low LD will not determine high RTC scores. Under H_1_, the RTC performs best in intervals with overall low LD, where the correlation between the eQTL and other non-disease SNPs decays much faster, making the dSNP correction stand out.

Finally, we investigated the effect of the overall LD pattern in a region of interest on the RTC. For this purpose, we calculated the median r^2^ of each hotspot interval and checked its relationship to the RTC score under the null and alternative hypothesis. It is expected that RTC will perform better in intervals with overall low LD, where the correlation between the eQTL and other non-disease SNPs will decay much faster, making the correction for the dSNP stand out. However, we confirm that the LD of the region does not determine high scores by itself. Intervals of low LD where different c-eQTLs and c-dSNPs reside have a uniform distribution of RTC scores (Figure 6, left panel). As expected, we do observe from the H_1_ simulations that we have most power in intervals with low median r^2^ (Figure 6, right panel).

### RTC scores when both traits are gene expression

As a positive control, we tested the method first on intervals harbouring already identified regulatory associations. We used published *cis* eQTLs (10^−3^ permutation threshold) discovered in the same tissue as the HapMap CEU eQTLs (LCLs) but derived from an independent set of samples: 75 individuals of Western European origin from the GenCord project [19]. In this experiment, we considered the GenCord eQTLs as the equivalent of GWAS SNPs and we limited our analysis to intervals with *cis* eQTLs in both datasets. Furthermore, we conditioned the associated genes for the same interval to be identical in the two expression datasets, expecting thus a common functional variant. As a result of this filtering, we tested SNPs in 157 hotspot intervals, associated with differential expression levels of 154 genes. As expected from the H_1_ simulations, the RTC Score distribution after correcting for the GenCord eQTLs is right-skewed (Figure 3, right panel), suggesting that the scoring method is sensitive to associations tagging the same functional variant. We detect 33 SNP-probe pairs with an RTC Score of 1 out of the total 185 tested pairs. Given the marked difference in genotyping density between HapMap and GenCord (∼1.2 millionSNPs versus ∼400,000 SNPs respectively) and our hypothesis that the 157 overlapping intervals share the same functional variant, we expect approximately 3 times more perfect scoring cases (99 pairs with RTC Score = 1) than what we observe, had individuals from both datasets been equally densely genotyped. We use the degree of sharing between the eQTLs in the two datasets to derive a reasonable, yet conservative threshold: currently, 105 SNP-probe pairs pass the 0.9 RTC threshold, making it thus a suitable stringent cut-off for calling significant discoveries.

### Significant causal *cis* effects

We then applied the scoring method on the NHGRI GWAS SNPs. The 976 common GWAS SNPs map to 784 hotspot intervals. Of these, we focused the *cis* analysis on GWAS intervals (N = 130) where at least one significant *cis* eQTL at a 0.05 permutation P-value threshold also resides (Dataset S1). For the trans analysis, we ordered all 784 GWAS intervals by their most significant trans eQTL and kept the topmost 50 intervals for further examination (Dataset S2). Table 1 summarizes our most confident *cis* results ordered by RTC Score. We detect SNP-gene combinations passing the 0.9 threshold for 28 intervals out of the 130, twice as many than expected by chance (13 expected top 10% scoring intervals under the uniform distribution). Our method confirms prior results in the literature suggestive of disease effects mediated through expression (ORMDL3 for asthma risk [13], C8orf13 locus for lupus risk [20], SLC22A5 for Crohn's disease [12],[21]). In addition, we detect several other yet unknown candidate genes for a variety of conditions.

**Table 1:** Candidate *cis* results.

GWAS SNP	Complex Trait	Gene	RTC	Chr
rs2064689	Crohn's disease	WDR78	1	1
rs3129934	Multiple sclerosis	HLA-DRB1	1	6
rs2188962	Crohn's disease	SLC22A5	1	5
rs1015362	Burning and freckling	TRPC4AP	1	20
rs2735839	Prostate cancer	C19orf48	1	19
rs6830062	Height	LCORL	1	4
rs2242330	Parkinsons disease	TMPRSS11A	1	4
rs7498665	Body mass index,Weight	EIF3CL	1	16
rs2872507	Crohn's disease	ZPBP2	0.99	17
rs255052	HDL cholesterol	AGRP	0.99	16
rs4549631	Height	TRMT11	0.98	6
rs9469220	Crohn's disease	ILMN_29412	0.98	6
rs11083846	Chronic lymphocytic leukemia	SLC8A2	0.98	19
rs13277113	Systemic lupus erythematosus	C8orf13	0.97	8
rs9272346	Type 1 diabetes	HLA-DRB1	0.96	6
rs12324805	Body mass index	STARD5	0.96	15
rs3764261	HDL cholesterol	MT1H	0.96	16
rs3135388	Multiple sclerosis	HLA-DRB5	0.96	6
rs3814219	Endothelial function traits	FAM26B	0.95	10
rs12708716	Type 1 diabetes	ILMN_32084	0.95	16
rs2269426	Plasma eosinophil count	HLA-DRB1	0.95	6
rs10769908	Body mass index	C11orf17	0.94	11
rs4130590	Bipolar disorder	ILMN_17339	0.94	9
rs7216389	Asthma	ORMDL3	0.94	17
rs3796619	Recombination rate (males)	CRIPAK	0.93	4
rs1748195	Triglycerides	DOCK7	0.93	1
rs2903692	Type 1 diabetes	ILMN_32084	0.93	16
rs3197999	Crohn's disease	SLC38A3	0.92	3
rs9858542	Crohn's disease	SLC38A3	0.92	3
rs6441961	Celiac disease	LIMD1	0.92	3
rs660895	Rheumatoid arthritis	PSMB9	0.91	6
rs9652490	Essential tremor	ILMN_111363	0.91	15
rs1397048	Hemostatic factors	OR8H2	0.91	11
rs3825932	Type 1 diabetes	CTSH	0.91	15
rs2395185	Ulcerative colitis	ILMN_29412	0.9	6

Candidate genes (RTC Score > = 0.9) for *cis* regulatory mediated GWAS effects. The higher the score, the more likely it is that the GWAS SNP and the eQTL for the gene shown are tagging the same functional variant.

An interesting example of a novel *cis* regulatory mediated effect is the one for Crohn's disease with gene SLC38A3, member 3 of the solute carrier family 38. Independent studies detected significant Crohn's associations of two SNPs in the same hotspot interval on chromosome 3 (rs3197999 [12], a non-synonymous SNP in gene MST1 and rs9858542 [1],[22], a synonymous SNP in nearby gene BSN). Suggestive literature evidence in addition to the disease associated non-synonymous SNP made MST1 the most attractive candidate gene out of the many present in that region [23]. However, our data supports an additional regulatory component underlying the susceptibility locus. For both GWAS SNPs, SLC38A3 is the highest scoring candidate in the region (RTC Score: 0.92). Interestingly, this is functionally similar to another Crohn's susceptibility gene SLC22A5 confirmed with our method (RTC Score: 1.0) and also encoding a sodium dependent multi-pass membrane protein (solute carrier family protein). The observed direction of effect is the same for both genes (eQTLs associate with low expression levels) as in previous expression datasets [12] and suggests a possible involvement of this gene family in the disease. This is in agreement with recent studies reporting that disease causative genes are functionally more closely related [24].

### Overrepresentation of immunity-related results

**Figure 7:**
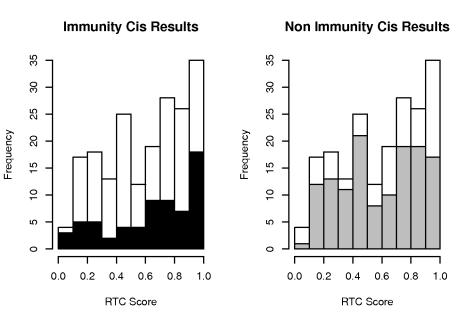
Overrepresentation of immunity-related high-scoring *cis* signals. Distribution of best RTC Scores per GWAS SNP stratified by immunity relatedness. Histogram contains results from the analysis of 130 hotspot intervals with colocalizing disease SNPs and *cis* eQTLs. We observe a significant overrepresentation of high-scoring (RTC > = 0.9) candidate genes (black bars) for immunity related complex traits compared to non-immunity related ones (grey bars) (Fisher's Exact Test, P-value = 0.0125).

The tissue under investigation is LCLs so we expect GWAS signals of immunity related traits (comprising here autoimmune disorders and diseases of the immune system e.g. AIDS progression) to more likely show an overlap with eQTLs. In order to evaluate the relevance of our results, we analyzed the distributions of the best RTC Scores per GWAS SNP stratified by the immunity relatedness of the complex trait they associate with (Figure 7). We observe a significant overrepresentation of high-scoring genes (> = 0.9) for immunity related traits compared to non-immunity related ones (Fisher's Exact Test, P-value = 0.0125) [25]. This suggests that the scoring scheme predicts regulatory effects of the relevant phenotypes. In addition, we observed that for GWAS signals with RTC score >0.9, only 10% of the nearest gene to the GWAS SNP was also the eQTL gene. These however, correspond as expected to instances when the eQTL gene is also the nearest gene to the eQTL itself. If that is not the case, the inference of relevance of a gene simply based on its proximity to the GWAS SNP is not informative.

### *Trans* effects

Even if the causal SNP is not *cis*-regulatory, using gene expression to determine its downstream targets, coupled with information about the biological pathways these targets act in could help interpret the primary GWAS effect. We investigated this hypothesis in the topmost 50 GWAS intervals ordered by their *trans* eQTL significance. For each interval, we apply the RTC Scoring scheme on the subset of genes in the whole genome with a notable effect in *trans* (SRC nominal P-value <10^−5^). These signals amount to a total of 552 genes. We obtain SNP-gene combinations passing the 0.9 Score threshold for 24 of the 50 tested intervals (corresponding to a total of 85 genes). Six of these intervals contain GWAS SNPs associated with immunity related traits (Table 2). While not statistically significant - unsurprisingly given that we're only testing a small subset of the total GWAS intervals - these examples support the usefulness of the *trans* approach. As hypothesized, for the same complex trait associated SNP we can discover several potential candidate genes in *trans*, throughout the genome. Some of these are biologically plausible results and merit further investigation. However, many *trans* candidates are hard to interpret at this stage given their incomplete annotation and further functional studies will need to be performed for validation.

**Table 2:** Candidate *trans* results.

GWAS SNP	Complex Trait	Genes	RTC	SNP Chr	Genes Chr
rs2251746	Serum IgE levels	SLC25A18	0.99	1	22
rs983332	Response to TNF antagonists	RGS16, IGSF3	0.97	1	1
rs983332	Response to TNF antagonists	C17orf58	0.97	1	17
rs653178	Celiac disease	PAX8, DOK1	1	12	2
rs17696736	Type 1 diabetes	PAX8, DOK1	0.98	12	2
rs2542151	Crohn's,Type 1 diabetes	MMP12	1	18	11
rs2542151	Crohn's,Type 1 diabetes	SLC39A4, PSD3, AHNAK2, FAM108B1, CYP2S1, CLEC7A	0.97	18	8, 8, 14, 9, 19, 12
rs2542151	Crohn's,Type 1 diabetes	LENEP	0.91	18	1
rs3134792	Psoriasis	ADRA2C	1	6	4
rs3134792	Psoriasis	DPEP1, ARHGEF3	0.99	6	16, 3
rs1265181	Psoriasis	POU5F1P1	0.96	6	8
rs1265181	Psoriasis	DPEP1	0.95	6	16
rs1265181	Psoriasis	CYP4F8, ADRA2C	0.94	6	19, 4
rs1265181	Psoriasis	RGS9	0.92	6	17
rs2395185	Ulcerative colitis	B4GALT2, ASB5	0.97	6	1, 4
rs2395185	Ulcerative colitis	STK32A	0.94	6	5
rs2395185	Ulcerative colitis	OXT	0.93	6	20
rs2395185	Ulcerative colitis	CSRP3	0.92	6	11
rs2395185	Ulcerative colitis	LGALS4	0.91	6	19
rs3135388	Multiple sclerosis	LIMS1	0.95	6	2
rs477515	Inflammatory bowel disease	B4GALT2	1	6	1
rs477515	Inflammatory bowel disease	ASB5	0.99	6	4
rs477515	Inflammatory bowel disease	STK32A	0.95	6	5
rs477515	Inflammatory bowel disease	OXT	0.94	6	20
rs477515	Inflammatory bowel disease	CSRP3	0.93	6	11
rs477515	Inflammatory bowel disease	DCHS2	0.91	6	4
rs477515	Inflammatory bowel disease	LGALS4	0.9	6	19
rs615672	Rheumatoid arthritis	DCHS2	0.99	6	4
rs6457617	Rheumatoid arthritis	SMARCD3	0.95	6	7
rs6457620	Rheumatoid arthritis	SMARCD3	0.95	6	7
rs660895	Rheumatoid arthritis	RETSAT	0.99	6	2
rs660895	Rheumatoid arthritis	CALCR	0.98	6	7
rs9268877	Ulcerative colitis	LIMS1	0.97	6	2
rs9268877	Ulcerative colitis	B4GALT2	0.94	6	1
rs9268877	Ulcerative colitis	ASB5	0.91	6	4
rs9272346	Type 1 diabetes	LIMS1	0.97	6	2
rs9272346	Type 1 diabetes	WHDC1L1	0.94	6	15
rs9272346	Type 1 diabetes	ASB5	0.93	6	4
rs9272346	Type 1 diabetes	SEMA6D, OXT, B4GALT2	0.92	6	15, 20, 1

Candidate *trans* genes likely involved in the same biological pathways, relevant to the GWAS SNPs. Signals relating to the same hotspot interval separated by a horizontal line. Table contains only the confident results (RTC Score > = 0.9) for the 6 immunity related intervals.

### RTC on GWAS data outperforms alternative correlation metrics

**Figure 8:**
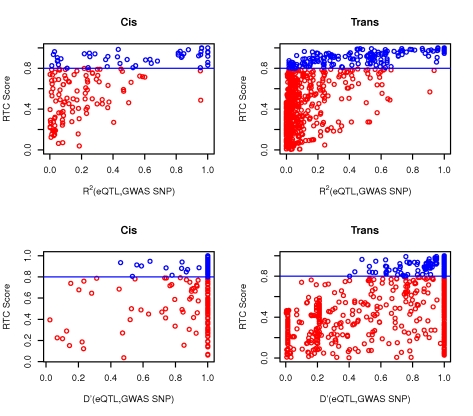
The RTC method compared to standard LD measurements in the observed data. Neither r^2^ nor D' between the eQTL and the GWAS SNP are direct predictors of a high RTC Score. Highlighted here are the results from the *cis* and *trans* analyses. We obtain high scoring results (RTC Scores > = 0.8 in blue) for cases with a high correlation between the disease SNP and the eQTL as expected, but also for pairs with low statistical correlation (r^2^ – top panel). As shown in the bottom panel, many of these high scoring pairs are historically correlated (D' = 1), but so are many more by chance. Additionally, we can detect high scoring pairs with low D' as well. Hence, no obvious combination of the two LD measures can predict a high RTC Score.

The power to detect significant associations between genotyped SNP proxies and a phenotype depends on the correlation between those proxies and the functional variant [26]. Just like for the simulated data, we tested whether the correlation between a GWAS SNP and its colocalizing eQTL is sufficient for predicting a shared causal effect. For both the *cis* and the *trans* analysis, we observe that the r^2^ between the eQTL and the disease SNP is not a direct predictor of the RTC Score, and in several cases we predict that even pairs with low r^2^ are likely tagging the same functional effect (Figure 8, top panel). The reason for this is that many of the high scoring pairs with poor statistical correlation (low r^2^) are actually historically correlated (D' = 1). Nevertheless, D' is not very informative either (Figure 8, bottom panel), the main problem here being that in regions with generally high D' among many SNPs, one cannot determine which of the pairs actually represents a common functional variant.

**Figure 9:**
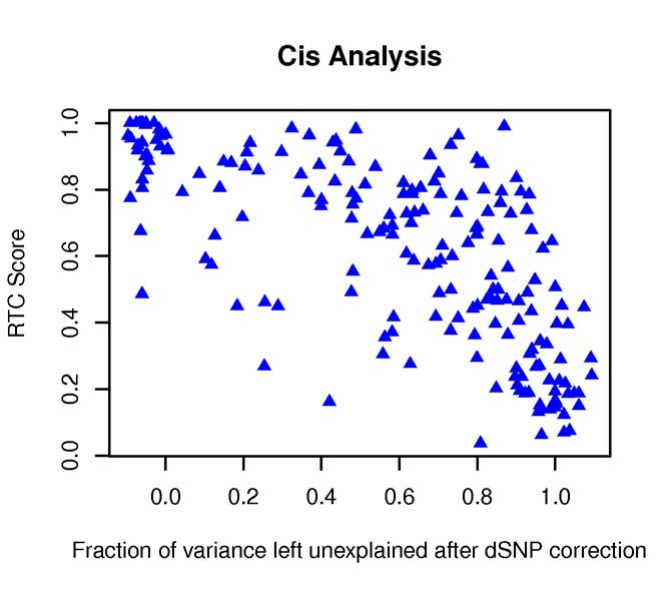
The fraction of eQTL variance explained away by the dSNP versus the RTC score. We contrast the LR adjusted R^2^ at the eQTL after and before correction of the dSNP and observe that while most high scoring pairs correspond to cases of lowest variance left unexplained, other interesting cases would be missed solely by using an arbitrary variance threshold.

Another metric of potential predictive value is the fraction of eQTL variance explained by the dSNP. Figure 9 indicates the relationship between the RTC score and the fraction of explained variance at the eQTL left unexplained after the dSNP correction (ratio of linear regression adjusted R∧2 after and before correction). As expected given the definition of the RTC, the highest density of good scoring results is registered for dSNPs that explain most of the eQTL variance. However, RTC outperforms the variance metric, scoring high even when that's not the case and thus making the setting of a threshold on the explained variance not sufficiently informative either.

## Discussion

To aid the functional interpretation of complex trait association signals, we describe here an empirical methodology that directly integrates eQTL and GWAS data while correcting for the local correlation structure in the human genome. As regulatory variants are pervasive throughout the genome, coincidental overlaps of eQTLs and GWAS SNPs are very likely. Hence, current methods that limit themselves to asking whether disease intervals also harbour eQTLs are unreliable for distinguishing trait relevant regulatory effects from other eQTLs. Our methodology addresses and helps resolve this issue.

This approach is not limited to gene expression, but could be generalized to any other phenotype. As new methods are developed and larger cohorts become available, various intermediate cellular phenotypes are interrogated via association studies with the hope to find explanatory links between genotypic variation and complex trait predisposition. However, the biological interpretation of these discoveries will also be hardened by the presence of tight LD. It is therefore necessary to evaluate them in a conservative manner, correcting for the local correlation structure in each genomic interval with overlapping association signals.

In this paper, we discover causal regulatory effects and their affected candidate genes in *cis* and to some extent in *trans* by assessing the impact on the expression phenotype of the removal of the GWAS SNP effect. We compute a score (RTC) for each individual genomic interval that assesses the likelihood that the eQTL and the GWAS SNP are tagging the same functional variant. By ranking the effect of the removal of the GWAS SNP in comparison to the outcome for any other SNP in the region and by accounting for the number of SNPs tested, we produce a score comparable across intervals. We evaluate the performance of the score in various simulated LD scenarios and we present its robustness by its expected uniform distribution when the eQTL and GWAS SNP are tagging different functional variants. In comparison, we investigate how well do current SNP correlation metrics (r^2^, D') perform on their own. We show that the LD between the GWAS SNP and its colocalizing eQTL is not a good predictor of a shared functional effect. This is very important especially since most of the current replication and follow-up studies only focus on variants highly correlated (r^2^>0.8) with the initial discoveries. It is important to stress at this point that neither the eQTL nor the GWAS SNP is likely the causal variant. Therefore, what really matters is not the statistical correlation between two proxies but the correlation between each of the proxies and the causal variant, whose frequency is unknown. In any case, no obvious combination of LD measures can substitute the RTC scoring scheme and we thus conclude that many interesting candidate genes would be missed if one were to rely solely on correlation-based approaches.

In this paper, we also explore the explanatory potential of regulatory variation given the currently published GWAS data. We observe a significant overrepresentation of eQTLs among GWAS SNPs, especially affecting genes in *cis*. Long-range *trans* effects are also present but less prevalent, possibly due to lower power to detect such associations. As expected given the tissue the expression data was measured in (LCLs), we observe a significant abundance of *cis* regulatory causal effects for immunity related traits. Our result reinforces the necessity to expand the tissue diversity [27]–[28] of genome-wide expression studies in order to facilitate such discoveries for a wider range of human conditions.

By applying the RTC method on the NHGRI GWAS SNPs, we are able to confirm previously suspected regulatory mediated disease effects and discover novel candidate genes affected by GWAS SNPs. We provide a list of follow-up candidate genes affected in *cis* and in addition, we show the utility of genome-wide expression data irrespective of the nature of the primary SNP effect by predicting clusters of genes affected in *trans*. The individual examination of the candidates prioritized with our approach will undoubtedly assist the biological interpretation of the ever-increasing list of GWAS signals. As associations with more intermediate cellular phenotypes will be reported, the integration of all these signals will be crucial for understanding the biology of complex traits.

## Methods

### Gene expression measurements

RNA levels were measured in lymphoblastoid cell lines (LCLs) derived from the HapMap 3 individuals using a whole-genome expression array (Illumina Sentrix WG-6, Version 2) as previously described [14]. Each sample had two technical replicates. We analyzed here only expression data from the CEU, a HapMap 3 population of 109 unrelated individuals of Northern European ancestry. The mapping of Illumina probes to unique Ensembl gene IDs resulted in 21,811 probes corresponding to 17,673 autosomal genes available for association analysis. 1,186,075 SNPs (MAF >5%) genotyped in the same individuals were used in the eQTL analysis.

### Post-experimental normalization of gene expression data

The log_2_ transformed raw intensity values were normalized as follows: quantile normalization of sample replicates (two intensity values per Illumina probe) followed by median normalization across all individuals.

### Genome-wide association study (GWAS) results

All SNPs from the catalogue of genome-wide association studies maintained by the National Human Genome Research Institute (NHGRI www.genome.gov/26525384) and published by 02.03.2009 were downloaded. Of these, only the 976 unique common variants (MAF >5%) genotyped in the HapMap 3 CEU samples were kept for analysis.

### Genotype-gene expression associations and multiple testing correction

Associations between SNP genotypes and normalized expression values were conducted using Spearman Rank Correlation (SRC). For the *cis* analysis, we considered only SNPs within a 1MB window from the TSS of genes, while in *trans* we test all SNPs further than 5MB away from the gene's TSS and all SNP-gene pairs on different chromosomes. We assess the statistical significance of the *cis* associations using permutations as previously described [7],[14]. We call a *cis* eQTL significant if the nominal association P-value is greater than the 0.01 tail of the minimal P-value distribution resulting from the SNP's associations with 10,000 permuted sets of expression values for each gene.

### Recombination hotspot interval mapping

We mapped all common autosomal CEU HapMap 3 SNPs (1,186,075 SNPs) to recombination hotspot intervals as defined by McVean et.al. [18] For the *cis* analysis we selected the 130 hotspot intervals where at least one significant *cis* eQTL and a GWAS SNP colocalize while for the *trans*, we analyzed a subset of 50 of the total 784 unique intervals (where the 976 GWAS SNPs map to). These are the topmost intervals ordered by their most significant *trans* eQTL (nominal SRC P-value).

### QQ plots of the abundance of regulatory signal in GWAS SNPs

For both the *cis* and *trans* GWAS analysis, the best P-value associations per SNP were stored. The set of the most significant P-values of the 976 GWAS SNPs was compared to 1000 sets of most significant P-values of 976 random SNPs. The 1000 random sets of 976 SNPs each were conditioned to have the same MAF distribution as the 976 GWAS set.

The QQ plot showing the abundance of regulatory signal in GWAS data is the median QQ plot of 1000 (GWAS, random SNPs) comparisons. It shows the distribution of the −log10 quantile values of the GWAS best associations (observed) versus the median of the corresponding 1000 −log10 quantile values from each of the 1000 random SNP sets (expected). In order to assess the significance of the observed versus expected median QQ plot, we superimpose the upper limit of the 95% confidence interval. This is calculated from the sorted 0.95 quantiles of 10000 pairs of 976 random SNPs each.

### Scoring scheme for determining causal regulatory effects

We assess the likelihood of a shared functional effect between a GWAS SNP and an eQTL by quantifying the change in the statistical significance of the eQTL after correcting for the genetic effect of the GWAS SNP. We redo the SRC association of the eQTL genotype with the residuals from the standard LR of the “corrected-for” SNP against normalized expression values. We account for the LD structure in each hotspot interval separately by ranking (Rank_GWAS SNP_) the impact on the eQTL (quantified by the adjusted association P-value after correction) of the GWAS SNP correction to that of correcting for all other SNPs in the same interval. By taking into account the total number of SNPs in the interval (N_SNPs_), we can compare this ranking across different genes and intervals. For this purpose we define the regulatory trait concordance (RTC) Score ranked below ranging from 0 to 1, with values closer to 1 indicating causal regulatory effects.
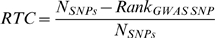



### Simulations of different causal SNPs (H_0_) and same causal SNP (H_1_) scenarios

We investigate the properties of the RTC score with respect to different correlation metrics under the null hypothesis (H_0_: eQTL and dSNP tag different functional variants) and the alternative hypothesis (H_1_: eQTL and dSNP tag the same functional variant).

We use the HapMap3 CEU *cis* eQTLs (315 genes at 10^−3^ permutation threshold) to create a list of causal SNPs (cSNP). For the H_0_, we call these cSNPs causal eQTL SNPs (c-eQTL). For each c-eQTL, we sample a different causal disease SNP (c-dSNP) from the same interval, with the requirement that its MAF comes from a distribution identical to that of the 976 NHGRI GWAS SNPs. Subsequently, we sample up to five eQTL-dSNP pairs per interval where the eQTLs and dSNPs are the topmost correlated (r^2^) SNPs with the c-eQTL and the c-dSNP respectively. After sampling, we exclude cases where the eQTL and dSNP are identical, as these contradict the H_0_..c-eQTL-c-dSNP-eQTL-dSNP quartets mapping to 287 unique hotspot intervals were sampled and tested under H_0_.

Under the H_1_, we sample up to five eQTL-dSNP pairs for each hotspot interval harbouring a cSNP as follows: the eQTLs are chosen as the top most significant SNPs per eQTL gene - excluding the cSNP; the dSNPs are randomly sampled from the same hotspot interval such that the r^2^ between each of them and the cSNP is in the range [0.5,0.9]. At any stage of the 5-step iteration per cSNP, the dSNP must be different from the cSNP and the eQTLs sampled up to that point. cSNP-eQTL-dSNP trios mapping to 290 unique hotspot intervals throughout the genome were sampled and tested under the H_1_.

We use the LD values (r^2^) of all pairwise SNP combinations per interval to calculate the median r^2^, an estimate of the LD extent per region.

### GenCord eQTLs as GWAS SNPs

To perform a control experiment where the trait is gene expression, we used *cis* eQTLs (10^−3^ P-value permutation threshold) detected in LCLs derived from 75 unrelated individuals of Western European origin from the GenCord project [19]. Hotspot intervals (N = 157) where both a HapMap and a GenCord eQTL associating with the same Ensembl gene reside were analysed with the RTC Scoring scheme.

## Supporting Information

Dataset S1 *Cis* analysis RTC results. Candidate *cis* regulatory effects as ranked by the RTC. No score filtering.
(0.08 MB XLS)

Dataset S2 *Trans* analysis RTC results. Candidate *trans* regulatory effects as ranked by the RTC in 50 genomic intervals. No score filtering.
(0.30 MB XLS)
